# An adult case of urinary tract infection with *Kingella kingae*: a case report

**DOI:** 10.1186/1752-1947-3-7236

**Published:** 2009-05-11

**Authors:** KV Ramana, SK Mohanty

**Affiliations:** 1 Department of Microbiology, Kamineni Institute of Medical Sciences (KIMS), Narketpally, Nalgonda-508254, India

## Abstract

**Introduction:**

*Kingella kingae*, though part of the normal upper respiratory tract and genitourinary tract, is increasingly being recognized as an important human pathogen. During the past decade, it has emerged as a significant pathogen in the pediatric age group primarily causing bacteremia and osteoarticular infections. Adult infection usually occurs in individuals who are severely immunocompromised and most infections have taken the form of septicemia or septic arthritis. Bacteremia due to *K. kingae* has been reported as the immediate cause of death in patients with acquired immunodeficiency syndrome.

**Case presentation:**

We present a microbiologically confirmed urinary tract infection with *K. kingae* in an immunocompetent 45-year-old adult woman with post-menopausal bleeding and with a history of clots. Her urine was subjected to culture and sensitivity tests. The isolated colonies were identified as *K. kingae* because of their typical culture characteristics such as long incubation period required for growth, beta-hemolysis, positive oxidase and negative catalase, urease indole, nitrate and citrate tests. Penicillin G disc test was positive. They were sensitive to all conventional antibiotics.

**Conclusion:**

*K. kingae* infection is a rare occurrence in immunocompetent adults. Very few cases of microbiologically confirmed infections have been reported so far. The isolation of *K. kingae* from urine sample has rarely been reported. *K. kingae* isolates are either missed or misinterpreted by clinical microbiologists. Therefore, *K. kingae* deserves recognition as a pathogen.

## Introduction

In 1976, *Moraxella kingae* was removed from the genus *Moraxella* and was given a new genus and species name *Kingella kingae* in the family *Neisseriaceae*[1]. Besides *K. kingae*, other species belonging to the genus *Kingella* are *K. denitrificans*, *K. indolegenes* and *K. oralis*. *K. kingae* exhibits a variable morphology (cocci, short Gram-negative coccobacilli to medium sized rods) and is considered to be a normal flora of the upper respiratory tract and genitourinary tract [2]. It has been associated with infections in children under 6 years and immunocompromised individuals [2].

Poor oral hygiene, pharyngitis and mucosal ulceration are the predisposing factors for *K. kingae* infections [2,3]. *K. kingae* bacteremia without endocarditis has also been reported in immunocompetent adults following dental manipulations [4]. *K. kingae* has specific tissue tropism for cardiac, valvular, joint space, and skeletal tissue and has been isolated from cases of bacteremia, endocarditis, bone and joint infection in various samples such as blood, joint fluid, and urine [5].

We report an adult patient with urinary tract infection from whom *K. kingae* has been isolated in urine.

## Case presentation

A 45-year-old woman was admitted to the gynaecology ward of Kamineni Institute of Medical Sciences Hospital, Narketpally complaining of post-menopausal bleeding with passing of clots for the previous 18 months. She also complained of burning micturition.

**Figure 1:**
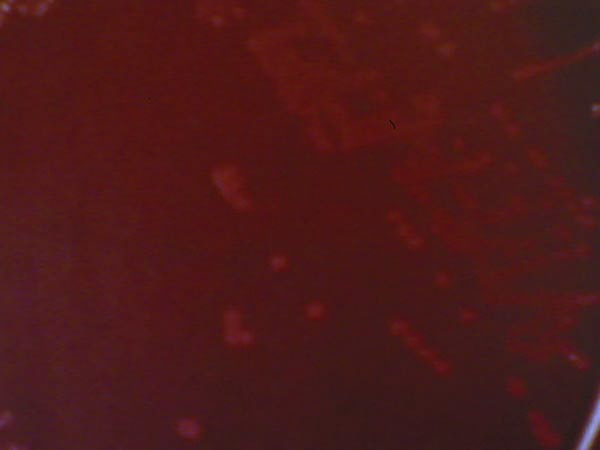
**Growth on Blood agar after 48 hours of aerobic incubation showing pinpoint translucent haemolytic colonies**.

**Figure 2:**
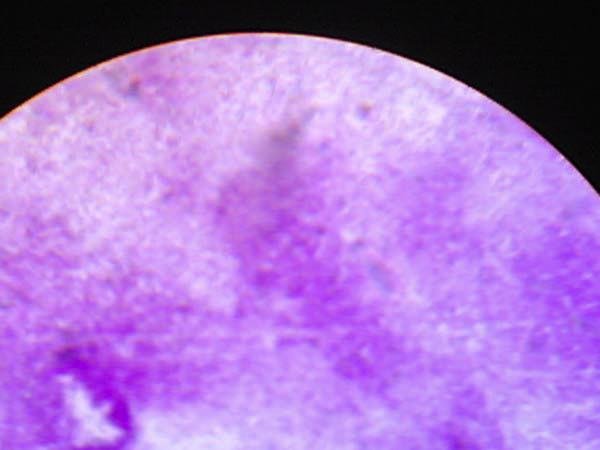
**Gram stain picture on day one showing Gram-negative cocci ' coccobacilli**.

**Figure 3:**
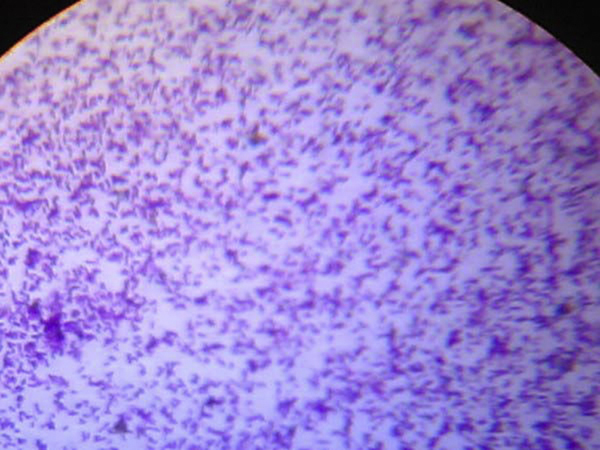
**Gram stain picture of first subculture showing Gram-negative bacilli**.

**Figure 4:**
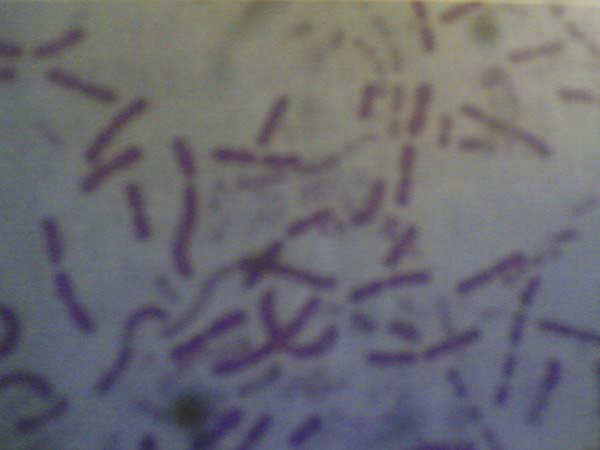
**Gram stain picture of colonies grown in the presence of penicillin (Penicillin G disc test) showing elongated Gram-negative bacilli**.

Urine was sent for culture and sensitivity tests. It was turbid and routine urine microscopic examination revealed the presence of 5 to 8 pus cells per high power field. Urine was inoculated in blood agar, MacConkey's agar and incubated at 37°C. Overnight incubation showed no growth. A pure growth of 1 mm round, convex β-hemolytic colonies was observed on blood agar after 48 hours of incubation, with no growth on MacConkey's agar (Figure 1). Gram stain of the isolated colonies showed varied morphology (cocci, diplococci, coccobacilli) (Figure 2). They were non-motile, oxidase-positive but were negative for catalase (*Moraxella* and *Neisseria* were positive), indole (*K. indolegenes* was positive), citrate, urease, and nitrate (*K. denitrificans* was positive). Only glucose and maltose (*K. oralis* was also positive for sucrose) were fermented. The Gram stained smear of the first subculture from blood agar showed short Gram-negative bacilli (Figure 3). Simultaneously, the Penicillin G disc test [6] was performed and revealed Gram-negative plump, elongated rods (*Neisseria* were negative) (Figure 4). The isolated colonies were found to be susceptible to penicillin (10 µg), ampicillin (25 µg), oxacillin (1 µg), gentamicin (30 µg), amikacin (30 µg), TMP-SMX (25 µg), cefotaxime (30 µg), ceftriaxone (30 µg), norfloxacin (10 µg), netilmicin (30 µg) and nalidixic acid (30 µg). The isolated organism was identified as *K. kingae* because of its fastidious nature, slow growth, β-hemolysis, varied Gram morphology, oxidase positivity, catalase negativity and positive penicillin G disc test.

## Discussion

*K. kingae*, though a part of the normal upper respiratory tract and genitourinary tract, is increasingly being recognized as an important human pathogen. During the past decade, it has emerged as a significant pathogen in the pediatric age group primarily causing bacteremia and osteoarticular infections [7,8]. Adult infection usually occurs in individuals who are severely immunocompromised and most infections have taken the form of septicemia or septic arthritis. Bacteremia due to *K. kingae* has been reported as the immediate cause of death in patients with acquired immunodeficiency syndrome (AIDS) [2].

*K. kingae* infection is a rare occurrence in immunocompetent adults. Very few cases of microbiologically confirmed infections have been reported so far [9]. The isolation of *K. kingae* from urine samples has rarely been reported [6]. We isolated *K. kingae* from the urine of an immunocompetent 45-year-old woman with post-menopausal bleeding and with clots for the previous 18 months. In this patient, the possible source of *K. kingae* was the resident genital flora. Due to the regular flow of clots and tissue, the organism could have gained access through the urethra causing ascending urinary tract infection. *Kingellae* are nutritionally fastidious Gram-negative bacilli requiring 48 hours of incubation before reaching a colony size of 1 mm diameter. They are oxidase-positive, catalase-negative (*Moraxella* are positive) and positive for the Penicillin G disc test. Of all of the species, *K. kingae* is β-hemolytic on sheep blood agar [2]. The isolation of *K. kingae* is often either missed or misinterpreted. Cases of *K. kingae* infection are on the rise in children as well as in immunocompromised and immunocompetent adults. Therefore, *K. kingae* deserves recognition as a pathogen.

## Abbreviations

AIDS: acquired immunodeficiency syndrome.

## Consent

Written informed consent was obtained from the patient for publication of this case report and any accompanying images. A copy of the written consent is available for review by the Editor-in-Chief of this journal.

## Competing interests

The authors declare that they have no competing interests.

## Authors' contributions

KVR analyzed and interpreted the patient data regarding the urine culture. SKM was a major contributor in writing the manuscript. Both authors read and approved the final manuscript.

## References

[B1] Henriksen SD Bovre K Transfer of Moraxella kingae Henriksen and Bøvre to the genus Kingella gen. nov. in the family Niesseriaceae Int J Syst Bacteriol 1976 26 447 450

[B2] Winn WC Allen S Janda WM Koneman EW Schreckenberger PC Procop G Baker Woods G Koneman's Color Atlas and Textbook of Diagnostic Microbiology 2006 6 Lippincott, Williams and Wilkins 472 473

[B3] Shimeld LA Rodgers AT Essentials of Diagnostic Microbiology 1999 Delmar Learning 180

[B4] Roiz MP Peralta FG Arjona R Kingella kingae bacteremia in an immunocompetent adult host J Clin Microbiol 1997 35 1916 9196226 10.1128/jcm.35.7.1916-1916.1997 PMC229874

[B5] Manuselis G Barnishan J Mahon CRManuselis G Textbook of Diagnostic Microbiology 2000 2 Saunders 440

[B6] Long KS Thomas JG Barnishan J Mahon CRManuselis G Textbook of Diagnostic Microbiology 2000 2 Saunders 408

[B7] Yagupsky P Dagan R Kingella kingae: An emerging cause of invasive infections in young children Clin Infect Dis 1997 24 860 866 9142783 10.1093/clinids/24.5.860

[B8] Yagupsky P Dagan R Howard CW Einhorn M Kassis I Simu A High prevalence of Kingella kingae in joint fluid from children with septic arthritis revealed by the BACTEC blood culture system J Clin Microbiol 1992 30 1278 1281 1583131 10.1128/jcm.30.5.1278-1281.1992 PMC265264

[B9] Van Damme PA Van Harpen CM Meis JF An adult case of oral infection with Kingella kingae Int J Oral Maxillofac Surg 2004 33 105 107 10.1054/ijom.2002.0440 14690666

